# Three monthly doses of 60 mg/kg praziquantel for Schistosoma *haematobium* infection is a safe and effective treatment regimen

**DOI:** 10.1186/s12879-020-05053-z

**Published:** 2020-05-06

**Authors:** Samuel Nkansah Darko, Henry Hanson, Sampson Twumasi-Ankrah, Sandra Baffour-Awuah, Priscilla Adjei-Kusi, Denis Yar, Ellis Owusu-Dabo

**Affiliations:** 1 Kumasi Centre for Collaborative Research in Tropical Medicine, Kumasi, Ghana; 2 Infectious Disease Epidemiology Research Group, Kumasi Centre for Collaborative Research in Tropical Medicine, Kumasi, Ghana; 3 Department of Mathematics, Kwame Nkrumah University of Science and Technology, Kumasi, Ghana; 4 Department of Basic and Applied Biology, University of Energy and Renewable Resources, Sunyani, Ghana; 5 Department of Science Education, University of Education, Winneba, Mampong Campus, Winneba, Ghana; 6 Department of Global and International Health, School of Public Health, Kwame Nkrumah University of Science and Technology, Kumasi, Ghana

**Keywords:** Praziquantel, Schistosomiasis, Resistance, Renal, Urinary

## Abstract

**Background:**

Praziquantel (PZQ) is the standard treatment for Schistosomiasis in sub-Saharan Africa. However, there is evidence suggesting praziquantel treatment failure in Schistosome infections with associated potential renal impairment. The objective of this study was to determine the effect of three monthly doses of 60 mg/kg/day PZQ on schistosome egg count, liver and renal function during the treatment of urinary schistosomiasis in Ghana.

**Methods:**

A nested case-control study was designed from a cohort screened for schistosomiasis; 28 schistosomiasis positive cases by microscopy matched with 53 healthy controls by age and gender. The study population was urban dwellers from the Asokwa sub-metropolitan area, Kumasi in Ghana. Participants were within the age range of 6 to 30 years. We assessed *Schistosoma haematobium* egg counts in urine and its associated impact on liver and renal function at baseline, treatment and post-treatment phases using serum.

**Results:**

Of the 28 cases and 53 controls, 78.6% and 81.1% were males respectively. Globulin levels before treatment was higher in cases [36.7 (32.8, 40.1) vrs 30.5 (22.4, 33.8), *p* = 0.005] at pre-treatment but not at post-treatment [35.8 (31.2, 39.1) vrs 37.4 (29.7, 43.0), *p* = 0.767]. Estimated cure rate was 42.9, 46.4 and 96.4% after first, second and third dose respectively. Schistosome egg counts dropped significantly (*p* = 0.001) from before second dose to post-treatment. Similarly, levels of alanine aminotransferase (*p* = 0.001), aspartate aminotransferase (*p* = 0.028) and gamma glutamyl transferase (*p* = 0.001) significantly declined towards post-treatment. Estimated glomerular filtration rate significantly improved from before second dose to post-treatment using both the Chronic Kidney Disease Epidemiology Program (*p* = 0.001) and 4-variable Modification of Diet in Renal Disease (*p* = 0.002) equations.

**Conclusion:**

Treatment of urinary *Schistosoma hematobium* infections with a repeated high monthly dose of 60 mg/kg of praziquantel for 3 months is safe and effective.

## Background

Praziquantel (PZQ) is virtually the sole treatment regimen for Schistosomiasis in sub-Saharan Africa [1]. This oral schistosomicidal agent is constituted of a racemate mixture, with activity both in vivo and in vitro [2, 3]. Evidence from studies conducted on *Schistosoma* (*S.*) *mansoni* and *S. japonicum* although not very clear, indicate the mode of action of PZQ is the targeting of calcium channels and antigen exposure rendering the worm susceptible to elimination by antibodies [1, 4].

After oral administration, PZQ is rapidly absorbed, metabolized and excreted by the kidney. Metabolism of PZQ is primarily via the cytochrome P450 system leading to the production of toxic metabolic intermediates, which are potentially harmful to hepatocytes [5]. Plasma levels of PZQ are also reported to be reduced by inducers but elevated by inhibitors of cytochrome P450 activity [6, 7].

Several studies, predominantly in Asian populations, where *S. japonicum* infections are endemic, state conflicting findings on hepatotoxicity associated with PZQ treatment against the helminth [8, 9]. PZQ treatment is reported to be associated with elevated serum concentrations of liver aminotransferase [8]. However, in a large retrospective study from China, there was insignificant (less than 1%) incidence of hepatotoxicity among populations treated for *S. japonicum* with PZQ [9].

Therapy for Schistosomiasis in sub-Saharan Africa has mainly been documented based on intestinal *S. mansoni* infections [1]. As a result, there is paucity of data on urinary *S. haematobium* and its associated drug metabolism effects on organs involved in metabolizing and excretion of PZQ. This leaves a gap in knowledge about the protective or destructive effect of metabolizing the drug in *S. haematobium* infection. It has further been shown that varied degrees of reduction in incidence and infection rates of *S. haematobium* are reported with mostly single PZQ dosage of 40 mg/kg/day in both children and adults [1]. There are also indications of drug resistance to single doses of PZQ for treating schistosomiasis [10]. This heightens the need to probe the outcome of repeated PZQ treatment on urinary schistosome counts against its implication on liver and renal function.

The aim of this study was to assess the effect of PZQ on schistosome egg count, liver and renal function after 3 doses of 60 mg/kg/day (PZQ60) in three months for treating urinary *S. haematobium* infection*.*

## Methods

This was a nested-Case Control study conducted among children and adults of ages 6–30 years from the urban Asokwa District in Kumasi, Ghana (see plate 1). This was part of a larger study to assess plasmodium transmission in persons infected with Schistosomiasis (NCT02769013). Ethical approval was obtained from the Committee for Human Research Publication and Ethics, School of Medical Sciences, Kwame Nkrumah University of Science and Technology, Ghana. All participants were required to sign an informed consent. For minors below 16 years, a signed assent form from the participant and an informed consent from a parent or guardian were obtained. Cases were respondents diagnosed to have *S. haematobium* by routine microscopic examination of urine samples. Controls from the same communities, without laboratory or clinical detection of urinary schistosomiasis infection were age and sex matched with cases.

### Study area

**Fig. 1:**
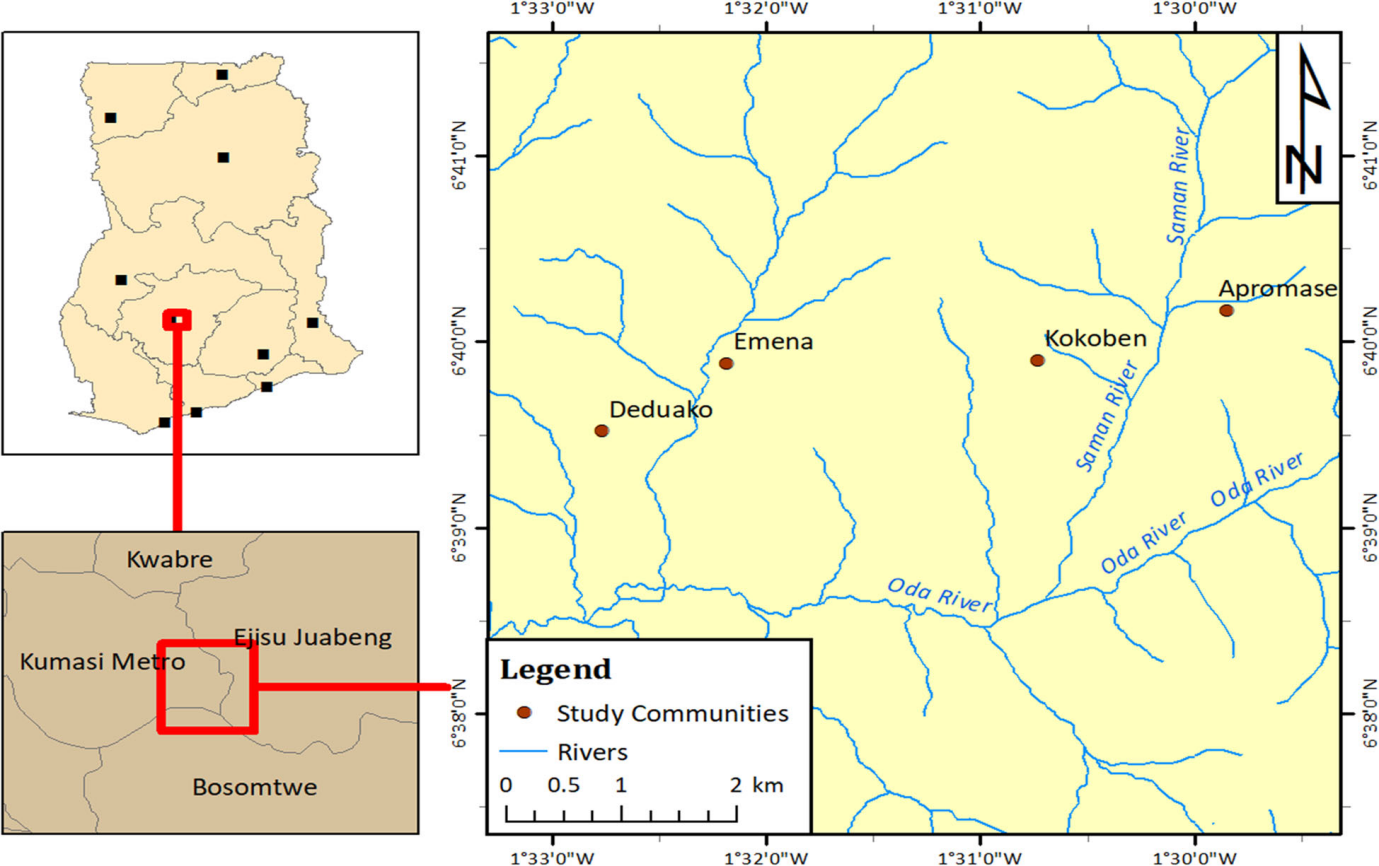
Map of study communities and sites (Rivers) in Ashanti region, Ghana

Apromase, Deduako, Emena and Kokoben were the study communities in the urban Asokwa District with a population of 140,161 inhabitants in 36, 183 households (Fig. 1) [11]. These communities are located between latitude 6°30′ and 7°00′ North and longitude 1°30′ and 2°00 West of Kumasi, the capital city of the Ashanti Region of Ghana. The four communities have Saman (Kokoben and Apromase), Oda (Deduako) and Subin (Emena) as names of three rivers running through it.

Climatic conditions are tropical with temperatures varying from 20.2 °C to 37.1 °C. Rainfall pattern is seasonally bimodal with major rains extending from late April to August with a minor one from September to October [12]. The average annual rainfall for the area is 6.25 mm with peaks of 214.3 mm and 16.2 mm in June and September respectively. The dry season (harmattan) is from November to March with humidity ranging between 53 and 93%.

### Screening and enrolment

A census of the selected communities was conducted with the ages and number of inhabitants per building collected along with corresponding GPS coordinates using Personal Digital Assistants (PDAs). Households within the buildings were selected and their members asked for written informed consent to be screened in the study. Twenty millilitres (20 ml) of urine samples were collected once, from consenting participant into well-labelled 30 ml urine containers. The urine samples were collected within the hours of 6:00 am and 12 noon. Subsequently, the samples were kept in cold boxes at temperature of 4–6 °C until transported to the laboratory at Kumasi Centre for Collaborative Research in Tropical Medicine (KCCR) about 15–20 min drive from the study sites.

Asymptomatic schistosomiasis positive (SP) cases and schistosomiasis-negative (SN) controls based on screening results were invited to participate in the study.

### Sampling procedures

**Fig. 2:**
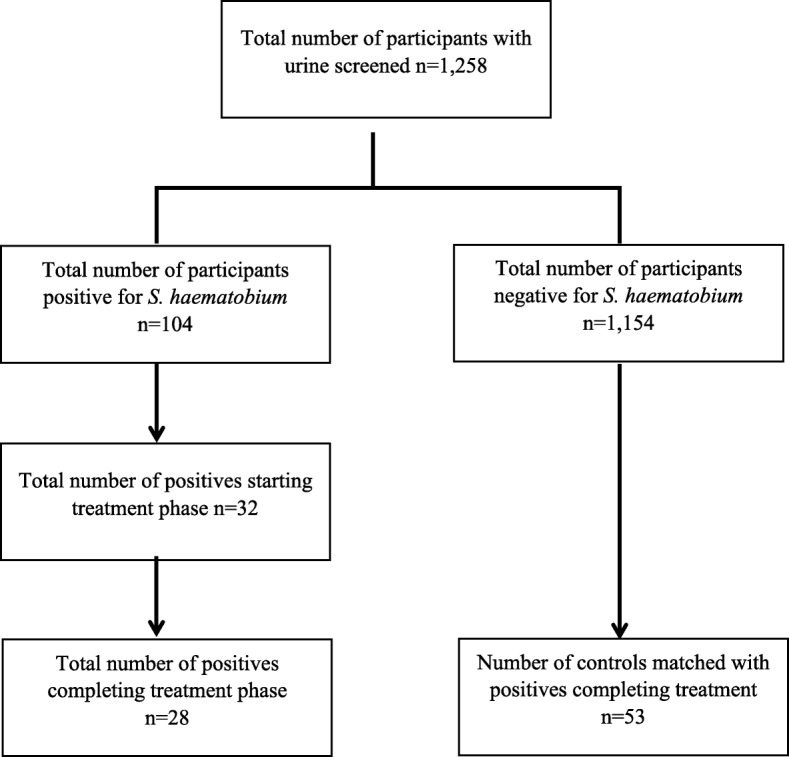
Sampling of study participants

A total of 1258 participants were screened for schistosomiasis out of which 104 were positive. All 104 schistosome positive participants (Fig. 2) were placed on PZQ60 treatment. Controls were selected from the same communities and matched in a 2: 1 ratio with cases by sex and age. Out of the 104 positive cases, 32 started the treatment phase with 28 successfully completing the course with samples analyzed. On the other hand, 53 controls had all samples analyzed.

### Design of Experiment

**Fig. 3:**
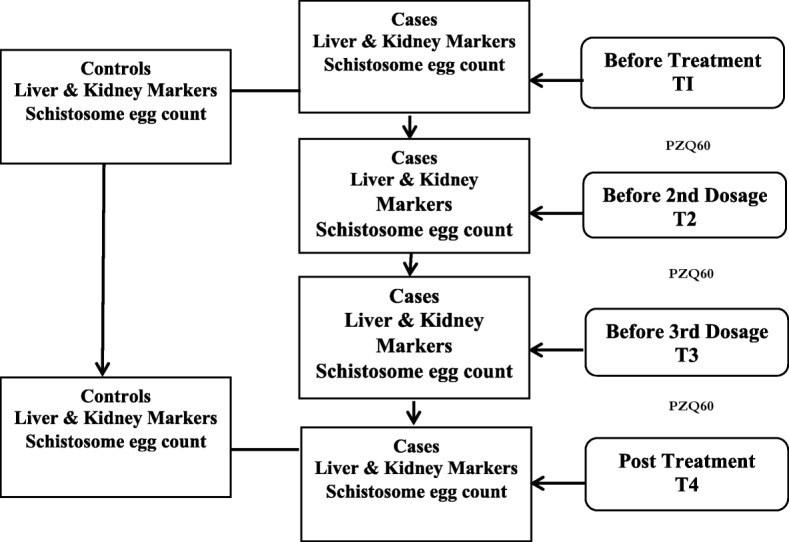
Flowchart of experimental design

Biochemical parameters and schistosome counts were analysed before and after treatment for both cases and controls (Fig. 3). In between pre- and post-treatment, the biochemical and schistosome counts were monitored before the 2nd and 3rd dosages of PZQ60 for cases.

### Laboratory processes

#### Processing of urine and S. haematobium quantification

Freshly voided urine was collected between 6:00 am and 12:00 pm in a sterile wide-mouthed screw-top plastic container (30 ml) and transported to the laboratory on ice at 4 °C to 8 °C. Urine processing and quantification were done as described by Cheesebrough [13]. Briefly, blunt-ended forceps were utilized to place a polycarbonate membrane filter of pore size of 12.0 μm (Whatmann Nuclepore) on the filter-support of a filter-holder (Swinnex 25 mm support chamber). The filter holder was re-assembled and attached to a 10 ml syringe filled with well-mixed urine which was filtered with the aid of the plunger. The filter was carefully removed and transferred with the face upwards to a clean glass-slide. A drop of physiological saline was added, covered with a cover-slip, and examined by two independent expert microscopists using the 10X objective (Carl Zeiss Microscope) with the condenser iris closed sufficiently to give good contrast. The entire filter was examined systematically for the presence of *S. haematobium* eggs. The number of the eggs counted per 10 ml of urine was recorded and the average of the two counts was calculated. A slide was declared negative when no parasites were detected.

#### Blood sampling and processing

Blood samples were collected from the antecubital vein with the aid of a tourniquet and the puncture site cleaned with 70% alcohol prep. Blood drawn into separator gel tubes (5 ml) were centrifuged at 1780 x g for 10 min at 4 °C to obtain the sera which were subsequently stored at -80 °C.

#### Biochemical analysis

Assays for the liver function; albumin, globulin, aspartate aminotransferase (AST), alanine aminotransferase (ALT) and gamma glutamyl transferase (GGT) and renal function (urea and creatinine), were conducted using a chemistry analyzer (HumaStar 200, Human, Germany). Elevated levels of AST, ALT and GGT are known indications of liver damage which may be caused by drug metabolism, infections and alcohol consumption. Increase in the levels of globulin is also found to correlate with infection or an inflammatory state. Aliquots of the serum were dispensed into cuvettes after thawing and placed at pre-programmed positions in the auto-analyzer and analysis done in batches. Due to the limited strength of serum urea and creatinine as markers of renal function, renal insufficiency based on estimated glomerular filtration was used as a more robust indicator. Renal insufficiency was assessed based on the 4-variable Modification of Diet in Renal Disease (4v-MDRD) and the Chronic Kidney Disease Epidemiology Program (CKDEPI) equations [14]. The 4v-MDRD estimates the glomerular filtration rate based on serum creatinine concentration, age, sex and race whiles the CKDEPI uses variations of an equation based on cut-offs for serum creatinine concentrations and sex.

### Statistical analysis

Data were entered into excel and analyzed with Stata V.12 (StataCorp, USA). Continuous variables were reported as median with interquartile ranges and categorical variables as proportions. The Wilcoxon signed rank test was used to assess differences in continuous variables between groups with statistical significance set at *p* < 0.05.

## Results

### Characteristics of study population before treatment

Majority of the study population were males [78.6 (62.4, 94.8) for schistosome positives and 81.1 (70.2, 92.0) for controls] with no significant statistical difference in age for the two groups (*p* = 0.547). Serum albumin levels were higher (*p* = 0.003) in controls [55.9 (52.1, 61.5)] compared with schistosome cases [51.9 (48.5, 53.0)]. On the contrary, serum globulin levels were elevated (*p* = 0.005) in cases 36.7 (32.8, 40.1) compared with controls 30.5 (22.4, 33.8). No significant difference was reported between schistosome cases and controls for liver (ALT, AST and GGT) and kidney (serum creatinine and urea) markers. Similarly, no significant difference (*p* = 0.600) was reported for estimated glomerular filtration rate (eGFR) between schistosome positive [96.0 (80.0, 116.0)] and negative [101.0 (88.0, 127.0)] groups using the CKDEPI equation before treatment (Table 1). Furthermore, the 4v-MDRD renal equation also showed no difference in levels between schistosome positive and negative groups at pre-treatment (*p* = 0.776).

**Table 1:** Demographics, schistosome counts and biochemical variables before treatment

Variable	Normal Ranges	Schistosome positive ***n*** = 28	Schistosome negative ***n*** = 53	***p*** value
**Male%**		78.6	81.1	
**Age (years)**		15.0 (12.5, 21.5)	16.0 (13.0, 22.0)	0.547
**Sh count (/10 mL)**		3.0 (2.0, 11.0)	0	0.033
**Albumin (g/L)**	35–55	51.9 (48.5, 53.0)	55.9 (52.1, 61.5)	0.003
**Globulin**	20–35	36.7 (32.8, 40.1)	30.5 (22.4, 33.8)	0.005
**Bilirubin (Direct) (**μmol**/L)**	0–5	5.7 (3.4, 7.5)	5.7 (4.5, 7.1)	0.825
**Bilirubin (Total) (μmol/L)**	3–22	14.1 (11.4, 20.2)	14.8 (12.4, 19.2)	0.809
**Creatinine (μmol/L)**	50–110	99.0 (77.0, 116.0)	91.0 (75.0, 114.0)	0.587
**GGT (U/L)**	9–48	20.0 (16.0, 26.0)	24.0 (16.0, 33.0)	0.243
**ALT (U/L)**	5–35	13.0 (9.0, 16.0)	13.0 (9.0, 18.0)	0.420
**AST (U/L)**	7–40	33.0 (28.0, 36.0)	30.0 (25.0, 35.0)	0.825
**AST/ALT ratio**	0.8	2.5 (1.9, 3.1)	2.6 (2.0, 3.1)	0.457
**Protein (g/L)**	60–80	89.0 (82.0, 92.0)	87.0 (80.0, 91.0)	0.840
**Urea (mmol/L)**	2.9–8.2	2.7 (2.1, 3.1)	2.6 (2.1, 3.0)	0.344
**eGFR (CKDEPI)**	> 90	96.0 (80.0, 116.0)	101.0 (88.0, 127.0)	0.600
**eGFR (4v-MDRD)**	> 90	100.5 (82.5, 120.0)	95.0 (83.0, 123.0)	0.776

*Sh S. haematobium* egg. eGFR (ml/min/1.73m^2^), proportion reported as percentage (interquartile range), biochemical variables reported as median (interquartile ranges)

### Effect of treatment of cases with 3 doses of PZQ60 on liver, renal function and S. *haematobium* egg count

**Fig. 4:**
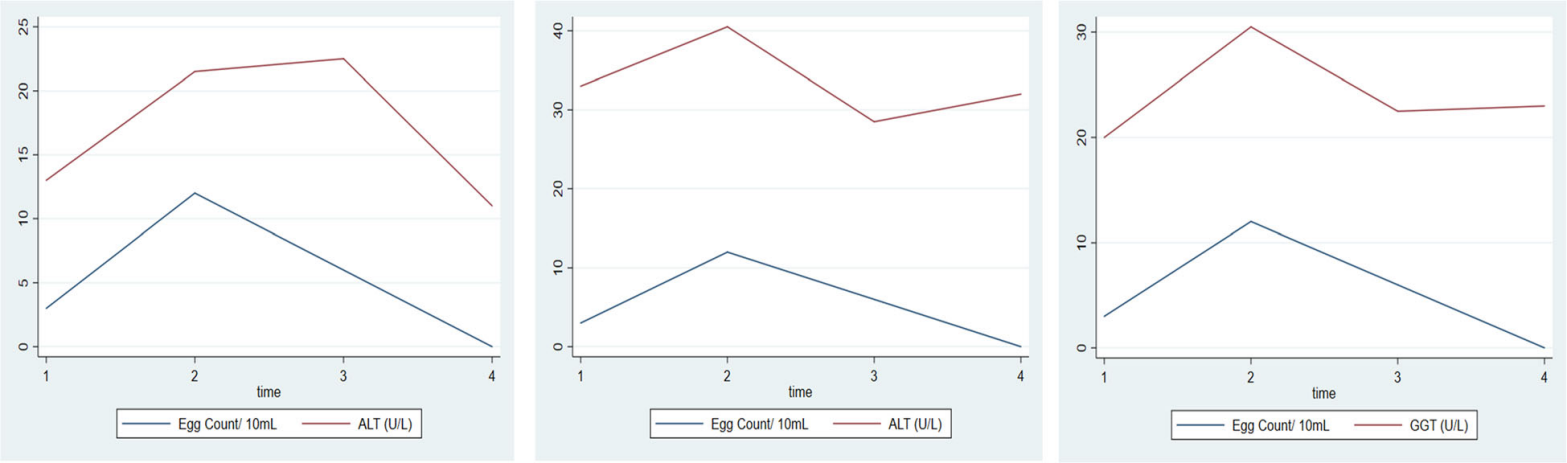
Effect of three repeated monthly doses of praziquantel 60 mg/kg on median liver enzymes and *S*. *haematobium* egg count. 1 = Pre-treatment, 2 = Before 2nd dose, 3 = Before 3rd dose, 4 = Post-treatment

After the first dose, ALT levels increased significantly (*p* = 0.001) and gradually declined after the second dose and then significantly (*p* = 0.001) towards post-treatment stage (Fig. 4). AST levels were elevated (*p* = 0.006) after the first dose and significantly (*p* = 0.001) declined before the third dose. However, there was no statistically significant difference (*p* = 0.577) in AST levels from before the third dose to post-treatment. GGT levels increased sharply (*p* = 0.001) after the first dose and declined significantly (*p* = 0.001) after the second dose. Schistosome egg count decreased significantly (*p* = 0.001) after the third dose towards post-treatment.

**Fig. 5:**
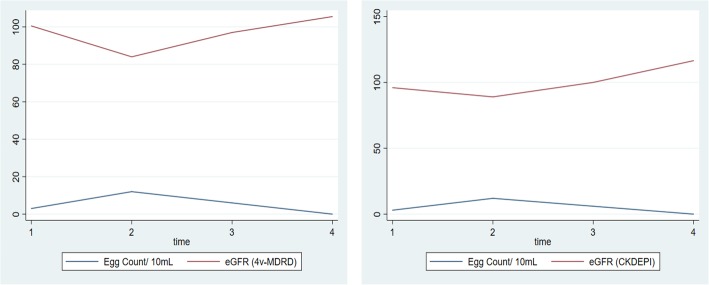
Effect of three repeated monthly doses of praziquantel 60 mg/kg on median estimated glomerular filtration rate and *S*. *haematobium* egg count. 1 = Pre-treatment, 2 = Before 2nd dose, 3 = Before 3rd dose, 4 = Post-treatment. eGFR reported in mL/min/1.73m^2^

Estimated glomerular filtration rates (Fig. 5) decreased significantly after the first dose using both CKDEPI (*p* = 0.003) and 4v-MDRD (*p* = 0.004). On the contrary, eGFR increased significantly from before second dose towards post-treatment for both CKDEPI (*p* = 0.001) and 4v-MDRD (*p* = 0.002).

### Effect of treatment with 3 doses of PZQ60 on cure rate

Estimated cure rate was 42.9% a month after administering the first dose of PZQ60 (Table 2). This increased to 46.4% before administering the third dose and finally to 96.4% post-treatment.

**Table 2:** Cure rates of 60 mg/kg praziquantel at stages of repeated dose treatment

	Before 2nd dose	Before 3rd dose	Post-treatment
**Total Sample**	28	28	28
**Median eggs/10 mL**	12.0 (1.0, 17.0)	6.0 (4.0, 38.0)	0
**Number cured**	12	13	27
**Cure rate**	42.9%	46.4%	96.4%

Median eggs reported with interquartile ranges

### Comparing Schistosome count and biochemical parameters between cases and controls after treatment

At post-treatment, there was no significant difference in mean levels for albumin (*p* = 0.441) between cases [48.8 (45.4, 52.5)] and controls [52.9 (49.6, 55.4)]. Similarly, mean levels of ALT, AST and GGT did not vary statistically between cases and controls post-treatment (Table 3). Moreover, creatinine and urea levels did not statistically vary between cases and controls. No significant difference (*p* = 0.753) was recorded using CKDEPI equation at post-treatment (Table 3) between the two groups. Likewise, 4v-MDRD reported no significant difference post-treatment (*p* = 0.866), between cases and controls.

**Table 3:** Schistosome count and biochemical parameters after treatment

Variable	Normal Range	Schistosome positive	Schistosome negative	***p*** value
**Sh count (/10 mL)**		0	0	
**Albumin (g/L)**	35–55	48.8 (45.4, 52.5)	52.9 (49.6, 55.4)	0.441
**Globulin**	20–35	35.8 (31.2, 39.1)	37.4 (29.7, 43.0)	0.767
**Bilirubin (Direct) (μmol/L)**	0–5	7.4 (5.8, 8.9)	7.0 (6.3, 8.0)	0.075
**Bilirubin (Total) (μmol/L)**	3–22	13.6 (11.1, 20.6)	14.2 (11.6, 16.7)	0.260
**Creatinine (μmol/L)**	50–110	91.0 (79.0, 111.0)	95.0 (77.0, 112.0)	0.441
**GGT (U/L)**	9–48	23.0 (20.0, 31.0)	26.0 (20.0, 39.0)	0.678
**ALT (U/L)**	5–35	11.0 (8.0, 13.0)	10.0 (9.0, 13.0)	0.513
**AST (U/L)**	7–40	32.0 (27.0, 35.0)	30.0 (26.0, 33.0)	0.722
**AST/ALT ratio**	0.8	2.8 (2.3, 3.4)	2.7 (2.1, 3.3)	0.441
**Protein (g/L)**	60–80	85.0 (77.0, 93.0)	85.0 (82.0, 96.0)	0.813
**Urea (mmol/L)**	2.9–8.2	2.5 (2.0, 2.8)	2.2 (1.9, 2.5)	0.635
**eGFR (CKDEPI)**	> 90	116.5 (94.0, 128.0)	94.5 (79.0, 115.0)	0.753
**eGFR (4v-MDRD)**	> 90	105.5 (87.0, 116.0)	90.0 (75.0, 109.0)	0.866

*Sh S. haematobium* egg, eGFR (ml/min/1.73m^2^), biochemical variables, ratio and eGFR reported as median (interquartile ranges)

## Discussion

Schistosome egg numbers declined after administration of the second dose of 60 mg/kg praziquantel and almost completely eliminated post-treatment with 96.4% cure rate. The estimated glomerular filtration rate dropped significantly after the first dose of praziquantel but resolved after the third dose. Moreover, levels of liver enzymes increased after the first dose and returned to pre-treatment levels in schistosome positive cases post-treatment.

Several studies assessing the efficacy of praziquantel treatment on *S*. *haematobium* has been based on single doses of 40 mg/kg with cure rates ranging from 39.8 to 88.9% in mainly children below 17 years [15–17]. In addition, multiple dose regimens have been reported to clear *S*. *haematobium* with 53.1 to 100.0% cure rates [18, 19]. We report a final cure rate of 96.4% after a monthly dose of PZQ60 for three months. Contrary to case reports [20, 21] where multiple courses of 40 mg/kg failed in clearing persistent *S*. *haematobium* infection, this study finding provides evidence of an efficacious treatment regimen with repeated and higher doses of PZQ60 which can clear Schistosome eggs and eliminate the worm stage of the parasite. This assertion is further supported by an initial increase in schistosome egg count before the second dose which could be attributed to a single dose of PZQ60 having little effect on juvenile worms as concluded in a previous study that persist and perpetuate more eggs [22]. It is worthy of note that although praziquantel is the standard treatment for schistosome infections due to reported efficacy and minimal side effects [23], there are studies that have found cases of drug failure [20, 21]. However, these investigations are not definite on the causes of these treatment failures. It is also plausible that the low cure rates after the first and second dose could be attributed to some level of resistance to PZQ previously reported in *S*. *haematobium* infections [24] in some endemic regions and could be now present in Ghana.

With respect to organ damage, many studies have tried to analyze the efficacy of varying concentrations of PZQ on organ injury [25, 26]. However, little is known of its effect to the liver and kidney as a result of repeated and higher doses for treating S. *haematobium* among sub-Saharan subjects. It is well established that metabolizing of PZQ analogs by cytochrome P450 can lead to highly oxidative intermediates [27] which overwhelm the body’s defense resulting in varied levels of organ damage. This study found a significant increase in liver enzymes after the first dose, which steadily dropped to pre-treatment levels. The initial upsurge in liver enzymes could be explained as due to highly oxidative intermediates of PZQ than can be quenched by the antioxidant activity of superoxide dismutase, glutathione and glutathione S-transferase. Consequently, hepatic cells are disrupted leading to the release of compartmentalized liver enzymes. However, the steady return after two more doses of PZQ to pre-treatment levels could be proof of the body system’s response of mopping up toxic metabolites by inducing higher expression of cells that synthesize these proteins involved in the body’s redox systems.

Similarly, the significant drop in estimated glomerular filtration rate for both the CKDEPI and 4v-MDRD equation after the first 60 mg/kg dose shows the body’s initial intolerance to oxidative species from the metabolism of the drug which improved even after monthly repeated doses. It is plausible that sustained higher thresholds of these induced protective antioxidants persist after its initial deficit compared with the highly oxidative drug intermediates associated with organ damage. This suggests possible protective effect of monthly repeated doses of PZQ on the renal filtration with a corresponding decline in schistosome egg count.

It is possible that taking other medications with this treatment regimen may further increase secretion of liver enzymes into blood. However, data backing this assertion is lacking from this study. Moreover, the reported levels of liver enzymes and estimated glomerular filtration rates could have been influenced by viral infections and wasting of muscles respectively. Even though the study finding gives a strong indication that PZQ60 given over 3 months is safe and effective, the willingness of only 32/104 (31%) of egg positive cases to participate in the repeated treatment (Fig. 2) suggests this regimen may have low compliance. It is recommended that further research is done on improving compliance to increase treatment coverage if multiple dosing is to be employed.

## Conclusion

This study suggests that treatment of urinary *S. hematobium* infections with a repeated high monthly dose of 60 mg/kg of praziquantel for 3 months is effective and safe. It provides an option to consider for *S*. *haematobium* infection cases with drug resistance.

## Data Availability

The datasets used and/or analysed during the current study are available from the corresponding author on reasonable request.

## Abbreviations

ALT: Alanine aminotransferase
AST: Aspartate aminotransferase
CKDEPI: Chronic Kidney Disease Epidemiology Program
eGFR: Estimated glomerular filtration rate
GGT: Gamma glutamytransferase
SN: Schistosomiasis negative
SP: Schistosomiasis positive
PZQ: Praziquantel
4v-MDRD: 4-variable Modification of Diet in Renal Disease
